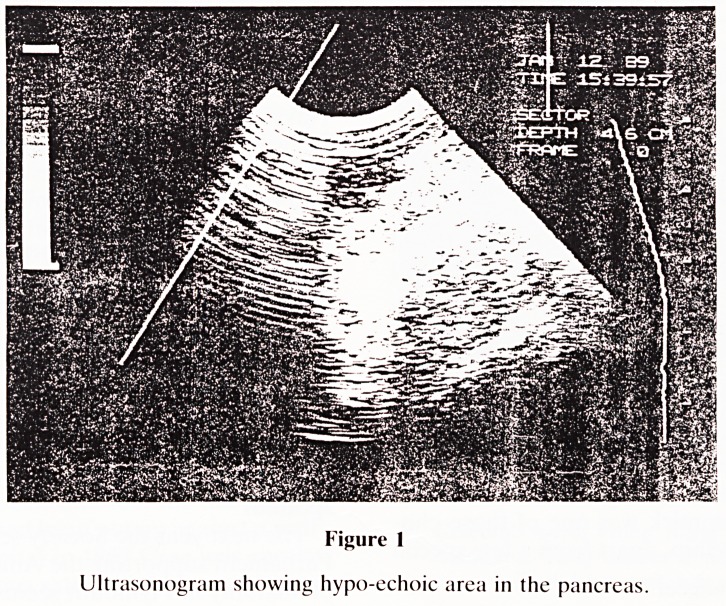# Intra-Operative Ultrasound in the Surgery of Insulinomas

**Published:** 1990-06

**Authors:** C. A. C. Clyne, W. J. Greene, R. B. Paisey

**Affiliations:** Departments of Surgery, X-ray and Medicine, Torbay Hospital, Lawes Bridge, Torquay, Devon TQ2 7AA; Departments of Surgery, X-ray and Medicine, Torbay Hospital, Lawes Bridge, Torquay, Devon TQ2 7AA; Departments of Surgery, X-ray and Medicine, Torbay Hospital, Lawes Bridge, Torquay, Devon TQ2 7AA


### Intra-operative Ultrasound in the Surgery of Insulinomas

**C. A. C. Clyne, W. J. Greene and R. B. Paisey**
Departments of Surgery, X-ray and Medicine, Torbay Hospital, Lawes Bridge, Torquay, Devon TQ2 7AA

Insulinoma is the commonest endocrine pancreatic tumour which causes profound effects due to hypoglycaemia. Successful excision is the treatment of choice. Pre-operative localisation is frequently difficult. CT scanning is of little use but percutaneous transhepatic portal vein catheterisation and venous sampling may be successful (1). Many patients are explored without pre-operative localisation and intraoperative ultrasound appears to be a simple and rapid adjunct to palpation in the detection of insulinomas.

### CASE HISTORY

A 66 year old woman presented (R.B.P.) with a 4 year history of "funny turns". She would suddenly feel faint, talk incoherently and feel dizzy. These attacks usually lasted less than 1 hour but on one occasion her symptoms of collapse necessitated her admission to hospital: her GP had found her blood sugar to be 2.3 mmol/l. She had generally enjoyed good health but 4 years previously had been referred to a neurologist for symptoms of transient diplopia and unsteadiness which had remained undiagnosed.

Examination was unremarkable and routine biochemistry and haematological tests were within normal limits. Fasting blood glucose samples of 1.7 mmol/l were associated with inappropriately high serum C—peptide and insulin levels (C-peptide 802 pmol/l; insulin 58 pmol/l) diagnostic of an insulinoma. Ultrasound, CT scanning and coeliac angiography failed to demonstrate the site of the tumour.

Operation was therefore undertaken (C.A.C.C.) through a transverse supra-umbilical incision. A full laparotomy was undertaken at which the only abnormality was the suggestion of a possible abnormal nodule in the pancreas at the junction of the body and head after exposue through the lesser sac.

Intraoperative ultrasound examination was performed using an ATL Mark 600 real-time scanner. The 7.5 MHz scanhead was protected by a sterile rubber sheath and the cable by a drill cover. A well-defined, roughly spherical 2 cm hypo-echoic mass was quickly identified at the location suspected on palpation (see Fig 1).

The tumour was easily enucleated and an insulinoma confirmed by frozen section. The abdomen was closed. The patient made an uneventful recovery although she required some insulin supplements for 48 hours. She was discharged home at 1 week and was seen at follow up 1 month later with no symptoms.

### DISCUSSION

Pancreatic insulinomas are notoriously difficult to localise pre-operatively with as many as 30 to 40% not having been visualised prior to laparotomy [1, 2]. Some have suggested that pre-operative localisation is unnecessary as the majority of tumours are easily found at surgery [3]. In each reported series, however, tumours are still not found in about 5% of instances. This must be regarded as less than optimum.

Intra-operative ultrasound is becoming more widely recognised as a valuable technique [4] and should certainly be available to any surgeon exploring the pancreas for insulinoma. In this way the detection rate of these tumours may be raised to nearly 100% [5].

### REFERENCES

1. AULD, C. D., BEASTALL, G. H., SCHLINKERT, R. T. and CARTER, D. C. (1988) *J. R. Coll. Surg Ed.* **33**, 132–137.
2. GORMAN. B., CHARBONEAU, J. W., JAMES, E. M., READING, C. C., GALIBER, A. K., GRANT, C. S., VAN HEERDEN, J. A., TELANDER, R. L. and SERVICE, F. J. (1986) Benign pancreatic insulinoma; Pre-operative and intra-operative sonographic localisation. *Am. J. Radiol.* 929–934.
3. DAGGETT, P. R., GOODBURN, E. A., KIRTZ, A. B., LE QUESNE, L. P., MORRIS, D. V., NABARRO, J. D. N. and RAPHAEL, M. J. (1981) Is pre-operative localisation of insulinomas necessary? *Lancet*, **28**, 483–486.
4. RIFKIN M. D., (Ed) Intraoperative and Endoscopic Ultrasonography; Churchill Livingstone, 1987.
5. LONDON, N. J. M., BOLIA, A., MILLAC, P., JAMES, R. F. L. and BELL, P. R. F. (1988) Localisation of an occult/impalpable insulinoma by intra-operative ultransonography. *J. R. Soc. Med.*, **81**, 663–664.

**Figure 1**

Ultrasonogram showing hypo-echoic area in the pancreas.

## Figures and Tables

**Figure 1 f1:**